# Accountability for reasonableness and criteria for admission, triage and discharge in intensive care units: an analysis of current ethical recommendations

**DOI:** 10.5935/0103-507X.20210004

**Published:** 2021

**Authors:** João Gabriel Rosa Ramos, Daniel Neves Forte

**Affiliations:** 1 Clínica Florence - Salvador (BA), Brazil.; 2 Intensive Care Unit, Hospital São Rafael - Salvador (BA), Brazil.; 3 Instituto de Pesquisa e Ensino D’OR - Salvador (BA), Brazil.; 4 Assistance, Teaching and Research Program in Palliative Care, Hospital Sírio-Libanês - São Paulo (SP), Brazil.

**Keywords:** Bioethics, Triage, Health care rationing, Noman Daniels, Intensive care units, Bioética, Triagem, Racionamento em saúde, Norman Daniels, Unidades de terapia intensiva

## Abstract

Triage for intensive care unit admission is a frequent event and is associated to worse clinical outcomes. The process of triage is variable and may be influenced by biases and prejudices, which could lead to potentially unfair decisions. The Brazilian Federal Council of Medicine (*Conselho Federal de Medicina*) has recently released a guideline for intensive care unit admission and discharge. The aim of this paper is to evaluate the ethical dilemmas related to the implementation of this guideline, through the accountability for reasonabless approach, known as A4R, as elaborated by Norman Daniels. We conclude that the guideline contemplates A4R conditions, but we observe that there is a need for indication of A4R-concordant criteria to operationalize the guidelines.

## Abstract (pt)

Triagem para admissão em unidades de terapia intensiva é um evento frequente, especialmente em situações de escassez de recursos, e está associada a piores desfechos clínicos. O processo de triagem é variável e pode ser guiado por vieses e preconceitos, levando à tomada de decisão potencialmente injusta. O Conselho Federal de Medicina elaborou recentemente uma resolução com os critérios de admissão e alta em unidades de terapia intensiva. O objetivo deste artigo é avaliar os dilemas éticos associados à implementação dessa resolução, tendo como prisma a abordagem do accountability for reasonableness (“responsabilização pela razoabilidade”), conhecido como A4R, conforme parâmetros elaborados por Norman Daniels. Apesar de a resolução em si contemplar as condições do A4R, ainda há espaço para que a norma indique critérios para que a operacionalização da resolução também contemple esses parâmetros.

## INTRODUCTION

Intensive care units (ICUs) aim to provide care to critically ill or high-risk patients.^(1)^ These units are able to provide sophisticated therapies and technologies, in addition to highly specialized human resources, and are potentially able to reduce the morbidity and mortality of critically ill patients in a cost-effective manner.^(2)^

However, scarcity of ICU beds is a global issue;^(3)^ thus, decisions about the allocation of resources are often made using triage and rationing.^(4)^ Empirical studies show that up to 51% of patients referred for ICU admission are not admitted; furthermore, this admission refusal is associated with higher hospital mortality,^(5)^ although the association may be subject to confounding factors because patients who are refused ICU admission are usually more severely ill than admitted patients.

In addition, there is evidence that potentially inappropriate ICU admissions are frequently triaged,^(6)^ which leads to nonbeneficial admissions to the unit or admissions that are inconsistent with the wishes and values of the patient and family members.^(7-9)^ This situation may have an impact on the direct medical care the patient receives and on the global allocation of resources, since inappropriate ICU admissions may be related to delays in the care of other patients.^(10,11)^

More worryingly, there is evidence that this triage process is variable^(12)^ and is affected by a number of patient factors,^(13)^ including nonclinical factors, such as personality,^(14)^ and structural factors,^(15)^ such as the number of available ICU beds.^(16)^ Thus, it is possible that current triage decisions are guided by biases and prejudices, which leads to potentially unfair decision-making. In addition, when the triage process itself is not structured, it can lead to distress and burnout for the health professionals involved.^(17,18)^

In an attempt to help guide these decisions, guidelines on the process of admission, triage and discharge in ICUs were created by medical societies and, recently, endorsed by the Brazilian Federal Council of Medicine (CFM - *Conselho Federal de Medicina*) in a resolution (number 2.156/2016).^(1)^ This resolution was based on the structure created by the guidelines of the Society of Critical Care Medicine^(18)^ and, among other objectives, was developed to provide a framework for the creation of specific institutional policies. Despite this effort, empirical studies show that health professionals may not adhere to these types of recommendations due to either a lack of knowledge or disagreement^(19)^ or practical difficulties with implementation.^(20)^

Considering that it is often not possible to reach a consensus on which underlying principles should guide triage decisions, a prioritization strategy based on a formal and fair process known as “accountability for reasonableness”, or A4R, has been proposed as an alternative to ensure a more equitable allocation of resources.^(21,22)^

In this work, we intend to evaluate resolution CFM 2.156/2016 from the perspective of the A4R prioritization strategy to verify whether the standard meets the necessary conditions for the establishment of priorities, as proposed by Norman Daniels.^(22)^ Before undertaking a specific evaluation of the standard, we will briefly introduce the work of Daniels, including the limitations of the classical models of distributive justice when applied to health.

## 1. Decisions on health rationing from the perspective of different conceptions of justice

Health systems have insufficient resources for health care; thus, prioritization decisions are made frequently and at different decision-making levels through hierarchical choices.^(4,23,24)^ It is likely that these decisions would still be necessary even if the resources to be spent on health increased and the efficiency of these expenditures was optimized.^(25)^ Decisions based on triage and rationing are necessary whenever a finite resource that is unable to meet all demands must be distributed, and it is necessary to define which needs should be prioritized to the detriment of others.^(25)^

For example, prioritization decisions are made at the macro and governmental levels, where budgetary decisions define which health strategies will be prioritized and whether health will be prioritized over other budget items.^(24-26)^ At the meso level, decisions about the allocation of resources within a health organization or about policies and guidelines for care in certain health situations direct the organization and the priorities of the services.^(4,24,25)^ For example, decisions regarding the purchase of equipment or the opening of new services, or even guidelines regarding which tests among a number of available possibilities should be requested in certain situations or which patients should receive priority care, impact the prioritization and rationing options in health systems. Finally, at the micro level, decisions are made by health professionals at bedside that are conditioned not only by decisions made in the higher hierarchical spheres but also by clinical judgment and the idiosyncrasies of the professional.^(4,24,26)^

There are few other contexts in which the impacts of prioritization decisions are as dramatic as in acute disease situations, in which they can have an immediate impact on the patient’s health or even his or her life.^(4,27)^

Such decisions raise a number of ethical issues since it is expected that health professionals respond to professional morality, which includes the obligation to intercede for their patients to ensure that they have access to the best available medical treatment.^(28,29)^ This moral obligation may conflict with the role of gatekeeper, i.e., the one who manages patient access to health services and treatments.^(24,30)^ Therefore, whenever possible, rationing decisions should be pre-established through collective solutions and with the participation of agents with an interest in the outcome.^(31)^

However, it is unlikely that all possible situations will be foreseen in collective solutions and, even if they are, the health professional him- or herself will be the agent who implements these decisions at the bedside, which requires interpretation and clinical judgment based on the immediate context.^(4)^ Thus, some form of ethical framework is necessary to assist health professionals in decision-making.

According to Fortes^(23)^ and others,^(26,28)^ some guidelines for a bioethical analysis of decisions regarding health resource distribution can be highlighted (Table 1). These guidelines are not necessarily mutually exclusive and can often be merged in the analysis of concrete cases.^(32)^ In addition to the justice orientations presented in table 1, others can be discussed, such as autocratic, democratic, luck-based and wellbeing-based conceptions.^(23,25,26,28,33)^

**Table 1:** Examples of distributive justice guidelines and their definitions

Distributive justice guidelines	Definitions
Justice based on contractual freedom	"The fundamental ethical principle to be observed in the organization of health systems is respect for the freedom of the human being to make decisions and make choices that affect his or her life".^(23)^ This guideline can be extended to the maxim "to each person according to the laws of the free market"
Justice based on strict equality and individual needs	The fundamental ethical principle is egalitarianism, i.e., "accepting that all people are worthy", which invokes equal access to resources. This guideline can be extended to the maxim "to each person according to his/her needs"^(23)^
Justice based on merit and social contribution	Differentiation of citizens to ensure that the distribution of resources takes into account "the natural endowments, their dignity, level of training and position in the organizational hierarchy"^(23)^
Justice based on social utility and maximization of benefits	Decisions and standards aim to promote the good and should therefore be evaluated by their results. The utilitarian principle provides that one should seek "the greatest happiness of all whose interests are at stake" and leads to the maxim "the greatest well-being for the greatest possible number of people"^(23)^
Justice based on equity	Provides for two basic principles. The first states that each person should be allowed the maximum amount of basic freedom compatible with a similar amount for all other people. The second principle states that social inequalities, although they may exist, must satisfy two conditions: they are only allowed if the global amount benefits everyone (for example, inequality arising from actions that lead to an enrichment of the population is seen as fair, although unequal) and if there is equal access to opportunities^(23,28,33)^

The presence of different distributive justice guidelines reflects a lack of consensus regarding the most relevant criteria for resolving conflicts that arise from the need to determine how to allocate scarce resources.^(22,34)^

According to Daniels,^(22)^ access to health is of particular moral importance due to its impact on the protection of access to opportunities, which is the fundamental basis of the theory of justice as equity, originally proposed by John Rawls.^(33)^ According to Paranhos et al.,^(35)^ by positioning health as fundamental for the promotion of equity, Daniels expands the original conception of Rawls based on the following proposals: (1) ensuring that health needs are met promotes health, (2) health promotes opportunity, so that (3) ensuring that health needs are met promotes opportunities.^(22)^

**Figure 1:**
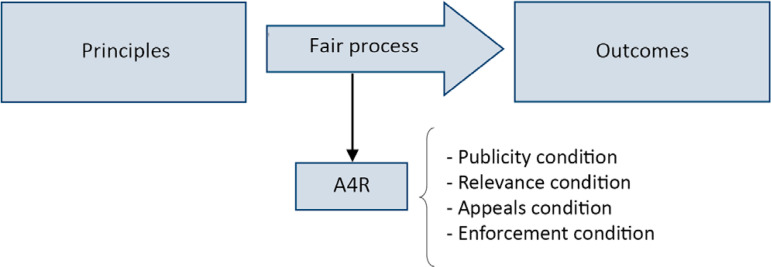
Diagram of the accountability for reasonableness approach.

According to the conception of Daniels, “in the absence of consensus on distribution principles, we need a fair process to establish legitimacy for critical resource allocation decisions”. In this author’s view, a fair process should address the problems related to legitimacy using the A4R ethical approach, as shown in figure 1.^(21,36)^

Most distributive justice guidelines focus on defining the principles or outcomes that should be prioritized. However, the accountability for reasonableness approach shifts the focus to the process, which must be formal, legitimate and fair and must respect the conditions of publicity, relevance, review and appeal and regulation. This assumes that it is very difficult (if not impossible) to reach a consensus regarding which principles and outcomes should be prioritized in all concrete cases.

A4R - accountability for reasonableness.

This approach was proposed to ensure robust public accountability regarding decisions related to triage and rationing^(22)^ by making the reasons or rationale behind these decisions publicly available. In addition, the reasons proposed in the approach are assumed to be those that impartial people would agree are relevant to appropriate patient care in a situation of resource scarcity.^(22)^ To ensure these principles, Daniels^(36)^ establishes four conditions that must be met within the A4R proposal:

1. Publicity condition: establishes that decisions about direct (or indirect) rationing and their rationales must be publicly available.
2. Relevance condition: establishes that rationales for rationing decisions must be reasonable; that is, this construct is considered reasonable if it is justified by the available evidence and if it appeals to reasons and principles that are considered relevant by people who “are willing to find mutually justifiable terms of cooperation”.^(36)^
3. Appeals condition: establishes that there should be mechanisms for disputing resolutions related to decisions regarding resource rationing and, more broadly, for ensuring opportunities for the review and improvement of policies if new evidence and new arguments emerge.
4. Enforcement condition: establishes that there must be regulation (public or voluntary) of the process to ensure that conditions 1 to 3 are met.

Thus, these principles make institutions responsible for the reasonableness of decisions related to resource rationing processes. Moreover, according to Friedman,^(37)^ Daniels is concerned not only with the actual legitimacy and equanimity of the process but also with the fact that the process must be perceived as legitimate, equitable and fair by the public. Nevertheless, the A4R approach is not free from criticism, and there are discussions about the democratization of discussions within this approach and the ability of A4R to establish the actual relevance of the rationales chosen for the prioritization systems, which would be of little help to the decision makers.^(37-39)^

However, the effort to establish priorities based on a fair process seems to be crucial in the attempt to resolve disagreements among experts. Thus, the A4R approach has been widely used in health systems.^(38)^ The application of this ethical approach has been evaluated both within and outside of ICUs,^(40-43)^ and the resulting studies show that the use of a process based on the A4R framework is generally perceived as fair and legitimate^(40,42)^ and can be used to identify good practices related to the establishment of priorities.^(41)^

## 2. Analysis of resolution 2.156/2016 of the Federal Council of Medicine

Resolution 2.156/2016 of the CFM established criteria for ICU admission and discharge and comprised 12 articles. Regarding resource rationing decisions, some articles of the resolution are of special importance, as follows (in free translation from Portuguese):

Art. 1 Admissions to intensive care units (ICU) should be based on:

I) diagnosis and patient need;

II) medical services available at the institution;

III) prioritization according to the patient’s condition;

IV) availability of beds;

V) potential benefit to the patient of therapeutic interventions and prognosis.

Art. 2 Admission and discharge from the ICU are within the responsibility and competence of the intensive care physician, considering the medical indication.

(...)

Art. 4 Patient admission and discharge from the ICU should be communicated to the family and/or legal guardian.

(...)

Art. 6 The prioritization of admission to the ICU must respect the following criteria:

§ 1 - Priority 1: Patients who require life support interventions, with high probability of recovery and without any therapeutic support limitation.

§ 2 - Priority 2: Patients who require intensive monitoring due to the high risk of requiring immediate intervention and without any therapeutic support limitation.

§ 3 - Priority 3: Patients who require life support interventions, with low probability of recovery or with limited therapeutic intervention.

§ 4 - Priority 4: Patients who require intensive monitoring because of the high risk of requiring immediate intervention, but with limited therapeutic intervention.

§ 5 - Priority 5: Patients who have a terminal illness, or are dying, with no possibility of recovery. In general, these patients are not suitable for ICU admission (except if they are potential organ donors). However, their admission may be justified on an exceptional basis, considering the peculiarities of the case and depending on the criterion of the intensive care physician.

(...)

Art. 9 Decisions regarding admission to and discharge from the ICU should be made explicitly, without discrimination based on religion, ethnicity, sex, nationality, color, sexual orientation, age, social status, political opinion, disability, or any other form of discrimination.

(...)

Art. 11. The ICU service of each hospital must develop protocols based on the criteria for hospitalization and discharge of this resolution that are in accordance with the specific needs of patients, considering the limitations of the hospital, such as ICU size and capacity for therapeutic interventions.

Sole paragraph. The ICU admission and discharge protocols should be disclosed by the clinical director to the hospital clinical staff and health system managers.^(1)^

The resolution also presents a statement of reasons in which the reporting councilor shows the rationale that led to the recommendations presented therein by making explicit that

ICU resources are limited and costly. Therefore, the occupation of ICU beds is essential and needs to be used rationally, which is complex and a great challenge, and that is why the establishment of clear criteria for patient admission and discharge from the ICU is justified.^(1)^

An analysis of the CFM resolution from the perspective of A4R can assess whether it includes the conditions assigned for the definition of a fair and equitable process.

1. Publicity condition: The CFM resolution is public, of unlimited access and was published in the *Diário Oficial da União* (Official Gazette) on November 17, 2016, Section I, p. 138-139, and is available on the websites of the Regional and Federal Councils of Medicine.
2. Relevance condition: Together with the explanatory memorandum, the resolution attempts to explicitly state the reasons for the recommendations made in its articles. The rapporteur establishes the reasons for ICU admission, the profiles of patients with potential to benefit, and potential alternatives for patients who have been refused an ICU bed.
3. Appeals condition: The resolution does not provide any mechanism for reviewing decisions that are based on it. The resolution states that the intensive care physician is responsible for making the decision based on medical indications, and it leaves no specific margin for discussion by other health professionals or patients/guardians. On the other hand, the normative functioning of CFM resolutions allows its internal standards to be revised through changes and revocations as new evidence and discussions emerge in society.
4. Enforcement condition: The CFM is an autarchy and is regulated by the judiciary and society in general (external regulation), as well as by the regional councils and their registered physicians (internal regulation).

Thus, it is shown that the resolution contemplates the necessary conditions to guarantee the principles of A4R. However, this resolution is a general recommendation of principles; it is not directly applicable and requires each service to create institutional protocols for the implementation of the standard, as defined in Art. 11. It is worth discussing, then, whether resolution 2.156/2016 of the CFM establishes appropriate tools for the implementation of its recommendations based on the principles of A4R.

## 3. Analysis of the recommendations for the implementation of resolution 2.156/2016 of the Federal Council of Medicine

Article 11 of CFM resolution 2.156/2016 addresses the implementation of its recommendations and establishes that institutional protocols should be applied by the ICUs of each service. It also notes that these protocols should consider the specific needs of patients and the limitations of the hospital. Finally, it provides that the protocols be disclosed to hospital clinical staff and health system managers.

By applying the component conditions of A4R logic, it is possible to analyze these recommendations.

### 3.1. Publicity condition in the implementation of resolution 2.156/2016 of the Federal Council of Medicine

Regarding the publicity condition, the resolution establishes that institutional protocols be made public for hospital medical staff and health system managers. By medical staff, we mean the physicians who work at the hospital in question.^(44)^ By health system managers, it is not clear whether the reference is to hospital directors or, in the case of the Unified Health System (SUS), to the municipal, state and federal spheres responsible for the direct or indirect management of the hospital in question. Specifically, when considering the supplementary health system, there is no reference to how protocols should be public to paying sources (health operators). In both cases (public and supplementary health systems), there is no clarity regarding the role of managers, whether consultative or deliberative. Specifically, it is not clear whether managers must approve relevant protocols or must only be informed of them.

More important, perhaps, is the fact that the parties with greatest interest in these policies - patients and their relatives - are not included among those who should have access to them. Although there is some discussion of the ethical nature of omitting specific details from discussions with patients if they are not likely to change any,^(45)^ this does not seem to apply in this case. Nevertheless, from the point of view of A4R, the condition of publicity is indispensable for ensuring accountability for the reasonableness of the process. This publicity is also essential as an attempt to reduce conflicts in cases of disagreement about the results of the ICU triage process in specific cases.

### 3.2. Relevance condition in the implementation of resolution 2.156/2016 of the Federal Council of Medicine

Regarding the relevance condition, the resolution establishes, in a generic way, that patient needs and hospital limitations are considered in the elaboration of the protocol. This recommendation, when associated with the recommendation of Art. 6, leads to the understanding that triage decisions for ICU admission should consider the following: (a) the availability of beds in the unit; (b) the need for admission to the ICU, read as the need for access to resources that can only be offered within this type of unit; (c) the probability of recovery; and (d) the presence of limitations of medical treatment.

It is clear that premise (a) is imperative. After all, one can only speak of triage in a situation in which the demand for a resource exceeds the available supply. Thus, triage decisions effectively take into account the availability of ICU beds, and there is a higher rate of admission refusal in situations in which beds in this unit are scarce.^(46)^ The focus is that the responsibility of the health professional does not necessarily end when the necessary resource is not available.

Premise (b) also seems to be rational. Since patients can be admitted to ICUs for intensive monitoring or life support interventions and intensive monitoring can potentially be made available in other sectors of the hospital, it seems logical that patients in need of ICU-specific interventions should have priority for admission to the unit. It is worth noting, however, that accepting this condition means accepting the underlying principle that the most severe patients should always be seen first, which is not necessarily universally accepted given that, in this resource allocation strategy, there is always a trade-off between dedicating more resources to acute patients and expanding the capacity to care for more subacute patients.^(47)^ By assuming that more severely ill patients should be treated first and less severely ill patients will not be harmed if they are seen in other units, such as semi-intensive care units, the resolution assumes a global maximization of benefit (greater number of lives saved) with this strategy. However, this strategy can generate controversy because there must be a balance between need (severity) and the probability of survival (benefit), especially in situations of absolute resource scarcity, to ensure that the greatest possible number of people receive care.

Premise (c), despite making *prima facie* sense, is complex to elaborate in practice. Health professionals have poor accuracy for predicting outcomes for critically ill patients, especially at the time of acute deterioration and ICU vacancy.^(48)^ Prognostic accuracy tends to increase with longer observation times, especially after the use of time-limited treatment attempts.^(49)^ However, these solutions may not be available in situations of extreme resource scarcity. The use of objective scores is a potential strategy to increase prognostic accuracy, but it is not risk-free.^(50)^ The use of probabilistic population scores to predict the potential benefits of ICU admission for individuals is difficult to interpret.^(51)^ Nevertheless, in the context of triage, it is less important to predict the probability a given patient’s survival than to predict the probability that intensive care will increase survival.^(52)^ In addition, the scores may not perform the same for the different pathologies of critically ill patients.^(52)^ As an example, the use of the Sequential Organ Failure Assessment (SOFA) to triage patients for admission to the ICU during the influenza pandemic^(53)^ would lead to the refusal of admission for patients who could potentially benefit from intervention.^(54)^

In addition, the very definition of what represents a high or low probability of recovery is not a consensus - even within the specialty of intensive medicine - since experts are not able to agree on a survival probability threshold that would be sufficient for admission or refusal to ICU admission.^(55)^ The underlying principle of allocating resources to those who are more likely to benefit (maximizing total utility) is also questionable, especially in situations where there is no clarity about what would be the fairest outcome and which benefit metric to use (survival, quality of life, functionality, work capacity, etc.).^(34)^

The use of expected benefit based on risk stratification models, especially when there is no clear definition of the objective parameters with which to define an “expected benefit”, may paradoxically run the risk of increasing injustice to specific groups.^(56)^ This can happen even when there is an increase in total utility (or expected total benefit, from the consequentialist point of view) because there may still be systematic negative consequences for the deprecated groups. This effect is known as “moral profiling”^(57)^ and may lead to discriminatory acts, incurring risks such as (1) stigmatization of groups with specific social or health situations (e.g., AIDS patients and homosexuals); (2) violation of privacy (due to the need to use sensitive personal data for risk stratification); (3) increased distributive injustice, since groups that already have some degree of disadvantage tend to be worse off and may be at risk of being denied access the specific resource (example: patients with difficulty accessing the health system who arrive at a hospital with greater severity of an acute disease and, therefore, less chance of recovery, may have a higher risk of not being admitted to the ICU, which would only increase the initial injustice); and (4) may endanger patient autonomy. It is worth noting that this risk is not mitigated by what is stated in Article 9, which is against any type of discrimination, since the discrimination exposed here is “statistical discrimination”; that is, it involves the differential treatment of people based on a characteristic, regardless of whether this differential treatment causes harm to the individual.^(57)^

Decisions regarding benefit and recovery can be subjective and variable,^(4,12,20)^ and thus, attempts to structure the decision-making process, such as defining the information that should be taken into account during decision-making^(58)^ or using decision support instruments in this process, are recommended.^(59)^

Premise (d) also makes sense initially since patients who will not use the monitoring and advanced treatments available in the ICU are unlikely to receive additional benefit from being hospitalized in this unit. However, this assumption reflects premise (c) and may thus incur similar risks.

### 3.3. Appeals condition in the implementation of resolution 2.156/2016 of the Federal Council of Medicine

The resolution does not standardize conditions for review or appeal in cases of conflicts between health professionals or between health professionals and family members.

The resolution states that admission to the ICU is a medical act and is the responsibility of the intensive care physician, but there is frequent disagreement regarding the appropriateness of ICU admission for specific patients, even among experts.^(12)^ Thus, it is possible to predict that there will be disagreements between physicians who request an ICU admission and those responsible for the admission decision. The resolution does not suggest or establish tools for resolving these disagreements, such as appeals to the technical director or the medical bioethics/ethics committee.

As discussed under condition 1, the resolution does not clearly require the participation of patients and guardians in decisions regarding triage for ICU admission, and Art. 4 establishes only that the decisions should be communicated to the patient and family members. In the explanatory memorandum, the rapporteur’s argument involves the perception of a high rate of inappropriate ICU admissions. This perception is corroborated by the medical literature.^(7)^ However, there is no consensus regarding what should be considered potentially inappropriate hospitalization.^(12,60)^

Futile treatments are those that are considered unable to achieve their physiological goals and that, therefore, should not be offered by health professionals.^(61)^ Potentially inappropriate treatments are those that are potentially capable of achieving their physiological goals but for which there are other ethical imperatives that may justify the non-administration of these treatments,^(62)^ most often because these treatments offer low expectations of out-of-hospital survival or neurological improvement and thus have limited perceived benefit.^(61)^ As the capacity for predicting the benefit of ICU admission is limited and there is no consensus regarding the prognostic threshold at which an ICU stay should be considered futile,^(55)^ in most situations, refused ICU admissions are considered potentially inappropriate and not futile.

It is noteworthy that in cases of potentially inappropriate treatments, it is preferable that the decision-making process be shared between health professionals and the patient/legal guardians by considering the available biomedical evidence, biography, values and wishes of the patient, the situational context and the deliberations of the two parties, with a focus on the objectives of care.^(63)^ The CFM itself^(64)^ establishes that, in the terminal phase of serious illnesses, the doctor is allowed to limit or suspend procedures and treatments that prolong the patient’s life while ensuring the necessary care to relieve symptoms that lead to suffering; however, they emphasize that this can only occur if it respects the will of the patient or his or her legal representative. This position was reinforced in Article 41 of the Code of Medical Ethics.^(65)^ Thus, treatments that are unable to achieve the goals of care defined for the patient, rather than the inappropriate use of health resources, are contrary to the ethical and professional values of nonmaleficence and respect for autonomy,^(66)^ since part of the nonmaleficence principle is the lack of a need to provide, or, according to some, the obligation to not provide, an ineffective treatment.^(67)^

Thus, it is clear that the refusal of ICU admission can only occur under usual conditions if it respects the wishes of the patient and his or her guardians. The resolution, however, makes no mention of decision-making or appeal processes in cases of conflicts or in unusual situations (such as catastrophic conditions),^(68)^ although other societies of medical specialties have created recommendations for this purpose.^(62)^ According to these recommendations, the approach to resolving these conflicts involves (1) consultations with negotiating experts to monitor the process, (2) making the decision-making process public for the patient and guardians, (3) obtaining a second medical opinion, (4) obtaining a review by an interdisciplinary hospital committee, (5) offering the possibility of transfer to another hospital service, (6) providing information about the possibility of extramural appeal (i.e., judicial) and (7) implementing the decision after process resolution.

The latter recommendations are especially important because when *prima facie* moral obligations are overcome by other obligations, they do not simply disappear; they leave a “moral residue”.^(28)^ When any agent performs an action that seems to be the best possible action under specific circumstances of conflict between different obligations, this agent may not be able to resolve all of the moral obligations related to the action; this leaves a “moral residue” that should be resolved later in various ways, such as through the resolution or prevention of other conflicts or through adequate compensation. In the case of triage for ICU admission, there may be a conflict between the principles of distributive justice and autonomy, and assurance that the best possible treatment alternative has been provided for patients who have been refused admission to the ICU must be part of the decision-making process. This ensures nonabandonment and continuity of care according to the objectives of care.

### 3.4. Enforcement condition in the implementation of resolution 2.156/2016 of the Federal Council of Medicine

The resolution does not establish standards to guarantee the enforcement condition of the process.

## CONCLUSION

Resolution 2.156/2016 of the Federal Council of Medicine, which establishes the criteria for intensive care unit admission and discharge, fills an important gap in a highly relevant topic. In general, the resolution *per se* seems to contemplate the conditions related to a fair and equitable process, as recommended by the A4R strategy. The resolution recommends the development of institutional protocols for implementing the recommendations, but there still seems to be room for improvement regarding the fair and effective implementation of the recommendations established in the resolution, according to the A4R paradigm. Specifically, there seem to be gaps in ways to (1) ensure the publication of institutional protocols for triage, admission and discharge from the intensive care unit; (2) establish an operational definition of the expected benefit of admission to the unit, with the aim of reducing the variability and subjectivity of the decision by defining the categories of variables to be identified or by using instruments to aid decision-making; (3) ensure respect for patient autonomy by making the patient part of the decision-making process; (4) structure the review and appeal process for intensive care unit admission decisions; and (5) establish a process (voluntary or external) for regulation of the protocol.
